# A stronger antibody response in increased disease severity of SARS-CoV-2

**DOI:** 10.1186/s12879-023-08923-4

**Published:** 2024-01-02

**Authors:** Marta Iglis de Oliveira, Melayne Rocha Aciole, Patrícia Areias Feitosa Neves, Vitor Palmares Oliveira e Silva, Marcelo Palmares Oliveira e Silva, Virginia Maria Barros de Lorena, Paulo Sérgio Ramos de Araújo

**Affiliations:** 1 Department of Tropical Medicine, Medical Sciences Center, Federal University of Pernambuco, Recife, Pernambuco Brazil; 2 Department of Immunology, Aggeu Magalhães Institute, Oswaldo Cruz Foundation, Fiocruz-PE, Recife, Pernambuco Brazil; 3 Department of Parasitology, Aggeu Magalhães Institute, Oswaldo Cruz Foundation, Fiocruz-PE, Recife, Pernambuco Brazil; 4 Department of Infectious Diseases, Hospital das Clínicas, Brazilian Company of Hospital Services (EBSERH), Federal University of Pernambuco, Recife, Pernambuco Brazil; 5 Recife Medical School, Medical Sciences Center, Federal University of Pernambuco, Recife, Pernambuco Brazil

**Keywords:** SARS-CoV-2, COVID-19, Antibody, Longitudinal study

## Abstract

**Background:**

An assessment of the factors that interfere with serum levels and the persistence of anti-SARs-CoV-2 IgG antibodies is essential in order to estimate the risk of reinfection and to plan vaccination. We analyzed the impact of the severity of coronavirus disease 2019 (COVID-19) and the clinical and biological factors regarding the persistence of SARs-CoV-2 anti-spike protein (IgG-S) antibodies at 12 months.

**Methods:**

This was an observational, longitudinal study with individuals who had recovered from COVID-19 between August 2020 and June 2021. Peripheral blood samples were collected from volunteers who were hospitalized (SERIOUS COVID-19) and those who required no hospitalization (COVID-19 LIGHT). Samples were grouped according to days after symptom onset: up to 90, between 91 and 180, ≥ 180 days after symptom onset. A semiquantitative test for IgG anti-spike protein S1(IgG-S1) was used.

**Results:**

We analyzed 238 individuals who had recovered from COVID-19, of whom 87 had been hospitalized and 151 had not. They provided 148 and 220 samples, respectively. Among those hospitalized, males (65.5%), volunteers aged over 60 years (41.1%), comorbidities such as arterial hypertension (67.8%) and diabetes mellitus (37.9%) were most frequent. We observed higher median serum IgG-S1 titers among those who had recovered from COVID-19 and had been hospitalized, at all collection time intervals (p < 0.001). We observed a weak correlation of increasing age with humoral IgG-S1 response (Spearman correlation = 0.298). There was a greater probability of IgG-S1 antibody persistence over time among samples from hospitalized individuals compared to samples from non-hospitalized participants (*p* = 0.001).

**Conclusion:**

This study has revealed higher titers and a higher probability of the persistence of IgG-S1 in severe cases after SARs-CoV-2 primary infection in unvaccinated recovered patients. Thus, in this study, the severe clinical presentation of COVID-19 was the main factor influencing serum levels and the persistence of IgG-S1 antibodies in COVID-19.

## Background

Since the beginning of the coronavirus disease 2019 (COVID-19) pandemic, efforts have been made to understand the kinetics of the anti-SARS-CoV-2 antibodies, particularly the duration of the levels of the immunoglobulin G (IgG) class, due to its protective role in reinfection [1–3]. Two hypotheses are of concern: the first is the decline in serum levels of IgG antibodies over time [4, 5] and the second is the greater chance of individuals with mild COVID-19 not seroconverting or generating a modest immune response compared to those with severe COVID-19 [6, 7].

Early studies have suggested a rapid decline in anti-spike IgG antibodies (IgG-S), the protein used by the coronavirus in order to enter cells, within three to six months after infection [8–10]. Subsequent data have demonstrated that IgG-S antibodies persist for up to eight months in non-hospitalized individuals [11, 12]. A prospective cohort with a 13-month follow-up period reported a significant drop in serum levels of IgG-S antibodies, and which occurred faster in males [3]. In the present study, it was not possible to verify an association with the severity of the disease, since only 4% of the individuals required hospitalization.

More recently, an assessment regarding how these molecules remain in the serum of individuals with the disease suggested that there are two types of anti-SARS-Cov-2 antibodies: antibodies with half-lives after 6 months and antibodies with half-lives for up to 14 months[13]. Most studies on humoral response in patients with severe COVID-19 have reported an association between disease severity and high antibody titers [14–16]. However, the short follow-up periods of these studies have not enabled the authors to assess the impact of COVID-19 severity on IgG antibodies that persist beyond 120 days.

The aim of this prospective study is to compare the serum levels and the longevity of IgG-S antibodies of individuals with severe and mild COVID-19, and to identify clinical and biological factors that may be associated with the dynamics of IgG-S antibodies in unvaccinated individuals who have recovered from COVID-19, after a primary infection with SARS-CoV-2 within 12 months of follow-up after symptom onset.

## Methods

A prospective, descriptive longitudinal study with an analytical feature was conducted in two high-complexity public hospitals in the city of Recife, Pernambuco, Brazil. Between August 2020 and June 2021, individuals were recruited aged over 18 years, who were treated at outpatient clinics for patients, who have recovered from COVID-19. Admission to the outpatient clinic occurred by referral from the attending physician after hospital discharge, by spontaneous demand or after an invitation by telephone contact from the research team. The study was approved by the institutional Ethics Committee of the Hospital das Clínicas at the Universidade Federal de Pernambuco (CEP-HC-UFPE 46681521.7.0000.8807).

At the first consultation, after signing the informed consent form, participants completed a questionnaire regarding sociodemographic characteristics, symptoms, comorbidities, and the need for hospitalization and were also submitted to peripheral blood collection. Those with no real-time polymerase chair reaction (RT-PCR) results or with a negative RT-PCR result for SARS-CoV-2 were excluded, as were individuals who had been vaccinated for COVID-19.

All RT-PCR tests for the detection of SARS-CoV-2 RNA, performed with nasopharyngeal swab samples, were analyzed at the Central Laboratory of Pernambuco (LACEN-PE).

Individuals were divided into two groups: Severe Cases: defined as those who required hospitalization; and Mild Cases: defined as those that did not require hospitalization. The Severe Cases group was further stratified according to oxygen demand: non-invasive oxygen therapy (nasal catheter and non-rebreathing mask) and invasive (invasive mechanical ventilation). The longevity and serum level of antibodies were assessed at three moments in time after the onset of symptoms: up to 90 days, between 91 and 180 days, after 180 days. Of the 238 individuals included in the study, 145 provided a single peripheral blood sample and 93 provided two or more samples.

For measuring the serum concentration of IgG-S1, the EUROIMMUN anti-SARS-CoV-2 ELISA kit (Lubeck, Germany) was used, one of the first diagnostic tests with the EC mark (European Conformity) to be developed, and available worldwide. The principle of this methodology is to quantify specific IgG to protein peak 1 (S1) of SARS-Cov-2 through an immunoenzymatic assay, where the results are presented in absorbance (optical density). This kit demonstrated a cumulative sensitivity of 82.6% for the detection of IgG in samples collected after 14 days of RT-PCR and a specificity of 86.9% [17]. To perform the test, the manufacturer's instructions were followed. The semi-quantitative test of the results was through the ratio between the absorbance level of the patient's sample by the absorbance level of the calibrator (Ratio). Ratio results < 0.8 were negative, ≥ 1.1 positive, and ≥ 0.8 and < 1.1 were indeterminate for the presence of IgG-S1.

### Statistical analysis

For the statistical analysis, SPSS 13 (Statistical Package for the Social Sciences) for Windows was used. The chi-squared test and the Kruskal–Wallis rank sum test were conducted to identify significant differences in the categorical variables between the groups. The Mann–Whitney test was used to compare quantitative data between the groups. All tests were two-tailed with a level of 0.05. Missing data were excluded for analysis. The odds ratio (OR) with a confidence interval was calculated.

The variables included in the univariate analysis were as follows: hospitalization, sex, age, arterial hypertension, type 2 diabetes mellitus (T2DM), obesity, IgG-S1 antibody titers up to 90 days, between 91–180, and after 180 days. Variables that attained a level of p < 0.2 in the univariate analysis were entered in a linear regression model using the Enter method.

A quality control of the models was performed: the assumption of linearity and the quality of variance of the dependent variable across the range of values of the independent variable were assessed with scatterplots, and the assumption that the dependent variable is normally distributed was assessed with a normal probability plot (data not shown).

The researchers were not blinded when recruiting participants nor when they assessed the results. However, the tests for detecting IgG-S1 were completely blind, since identifying the biological samples did not define the stratification of the groups. Only the final analysis of the data revealed an overview of the results. Potential confounders were identified and controlled in the data analysis.

## Results

**Fig. 1:**
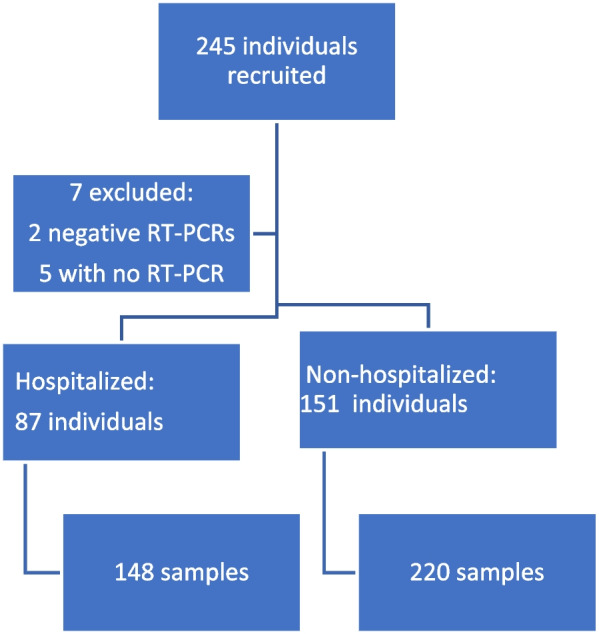
Screening, Enrollment and Stratification. Among the recruited, seven were excluded for having presented a negative molecular test or for failing to present test results. 238 participants provided 368 samples over 386 days after symptom onset. RT-PCR denotes Real-time polymerase chain reaction

A total of 245 individuals were referred to the outpatient clinic for those who have recovered from COVID-19, of whom, two were excluded for having presented a negative molecular test, and five were excluded for failing to present test results. Thus, 238 individuals were included in the study, who contributed 368 samples. The 87 participants who had been hospitalized provided 148 peripheral blood samples and the 151 participants who had not been hospitalized provided 220 peripheral blood samples over 386 days after symptom onset (Fig. 1).

## Clinical and biological characteristics of individuals recovered from COVID-19 according to hospitalization (Table 1)

**Table 1:** Clinical and biological characteristics of 238 individuals who recovered from COVID-19 according to hospitalization, in 386 days after primary infection

**Variables**	**Hospitalization**				
	**Yes**	**No**	**OR**	**CI 95% OR**	***p*** **-value**
	***n*** ** = 87 (%)**	***n*** ** = 151(%)**			
**Sex**					
Male	57 (65.5%)	73 (48.3%)	2.03	1.18 – 3.50	0.010 *
Female	30 (34.5%)	78 (51.7%)	1.00	–-	
**Ages (in years)**					
18 to 45	14 (16.1%)	67 (44.4%)	1.00	–-	< 0.001*
46 to 59	37 (42.5%)	65 (43.0%)	2.72	1.35 – 5.50	
≥ 60	36 (41.1%)	19 (12.6%)	9.07	4.07 – 20.19	
**Symptoms**					
Fever	61 (70.1%)	74 (50.0%)	2.35	1.34 – 4.11	0.003*
Cough	64 (73.6%)	72 (48.6%)	2.94	1.65 – 5.22	< 0.001*
Dyspnea	74 (85.1%)	46 (31.1%)	12.62	6.37 – 25.03	< 0.001*
No change in sense of smell	48 (55.2%)	49 (33.1%)	2.49	1.14 – 4.28	< 0.001*
Myalgia	40 (46.0%)	74 (50.0%)	0.85	0.50 – 1.45	0.551*
Asthenia	45 (51.7%)	84 (56.8%)	0.82	0.48 – 1.39	0.454*
Sore throat	11 (12.6%)	51 (34.5%)	0.28	0.13 – 0.56	< 0.001*
Diarrhea	18 (20.7%)	46 (31.1%)	0.58	0.31 – 1.08	0.084*
Abdominal pains	10 (11.5%)	19 (12.8%)	0.88	0.39 – 1.99	0.762*
Headache	28 (32.2%)	100 (67.6%)	0.23	0.13 – 0.40	< 0.001*
**No symptoms**	0 (0.0)	11 (7.3%)	–-	–-	–-
**Comorbidities**					
SAH	59 (67.8%)	35 (23.8%)	6.74	3.74 – 12.15	< 0.001*
T2DM	33 (37.9%)	13 (8.8%)	6.30	3.08 – 12.88	< 0.001*
Obesity	38 (46.3%)	50 (36.2%)	1.52	0.87 – 2.64	0.139*
**Oxygen therapy**					
Non-invasive	69 (79.3%)	–-	–-	–-	–-
Invasive mechanical ventilation	18 (20.7%)	–-	–-	–-	–-
**Time in ICU**	29 (33.3%)	–-	–-	–-	–-
**Distribution of samples**					
Up to 90 DASO	72 (48.6%)	75 (34.1%)	–-	–-	0.003*
91 to 180 DASO	41 (27.7%)	58 (26.4%)	–-	–-	-
> 180 DASO	35 (23.6%)	87 (39.5%)	–-	–-	-
**Presence of IgG-S antibody**					
Positive	145 (98.0%)	168 (76.4%)	11.22	3.40 – 37.07	< 0.001*
Negative	3 (2.0%)	39 (17.7%)	1.00	–-	
Undetermined	0 (0.0)	13 (5.9%)	**	**	

(*) Chi-Squared (**) Incalculable

SAH denotes Systemic arterial hypertension, T2DM Type 2 diabetes mellitus, ICU intensive Care Unit, DASO Days after symptom onset, IgG-S Anti-spike Immunoglulin G, OR Odds Ratio

This table shows the frequency of clinical and biological characteristics between hospitalized and non-hospitalized groups. Using Chi-Squared test, significant differences between the groups were identified. We also report the Odds Ratio with a corresponding 95% confidence interval to estimate the chance of each variable occurring in the hospitalized group compared to non-hospitalized group

The most frequently hospitalized patients were male (65.5%, *p* = 0.01). In the hospitalized group, 36 (41.1%) patients were aged 60 years or older (< 0.001). Among the 151 non-hospitalized participants, symptoms such as smell or taste changes, sore throat and headache were significantly more frequent. On the other hand, fever, cough and dyspnea were more common in the hospitalized group.

The presence of specific IgG-S1 antibodies against SARS-CoV-2 was verified in 313 samples. Forty-two samples were classified as negative for specific IgG-S1 antibodies against SARS-CoV-2. Of these, 39 samples were from non-hospitalized patients who had recovered and three samples were from hospitalized patients who had recovered.

Comorbidities such as systemic arterial hypertension (SAH) and diabetes mellitus were more prevalent among hospitalized patients (p < 0.001). All hospitalized participants had received oxygen therapy. Among these, 69 (79.3%) received non-invasive oxygen therapy and 18 (20.7%) received invasive ventilatory support and 29 (33.3%) were admitted to an intensive care unit.

### Factors that impact the response of specific IgG-S1 against SARS-CoV-2

**Fig. 2:**
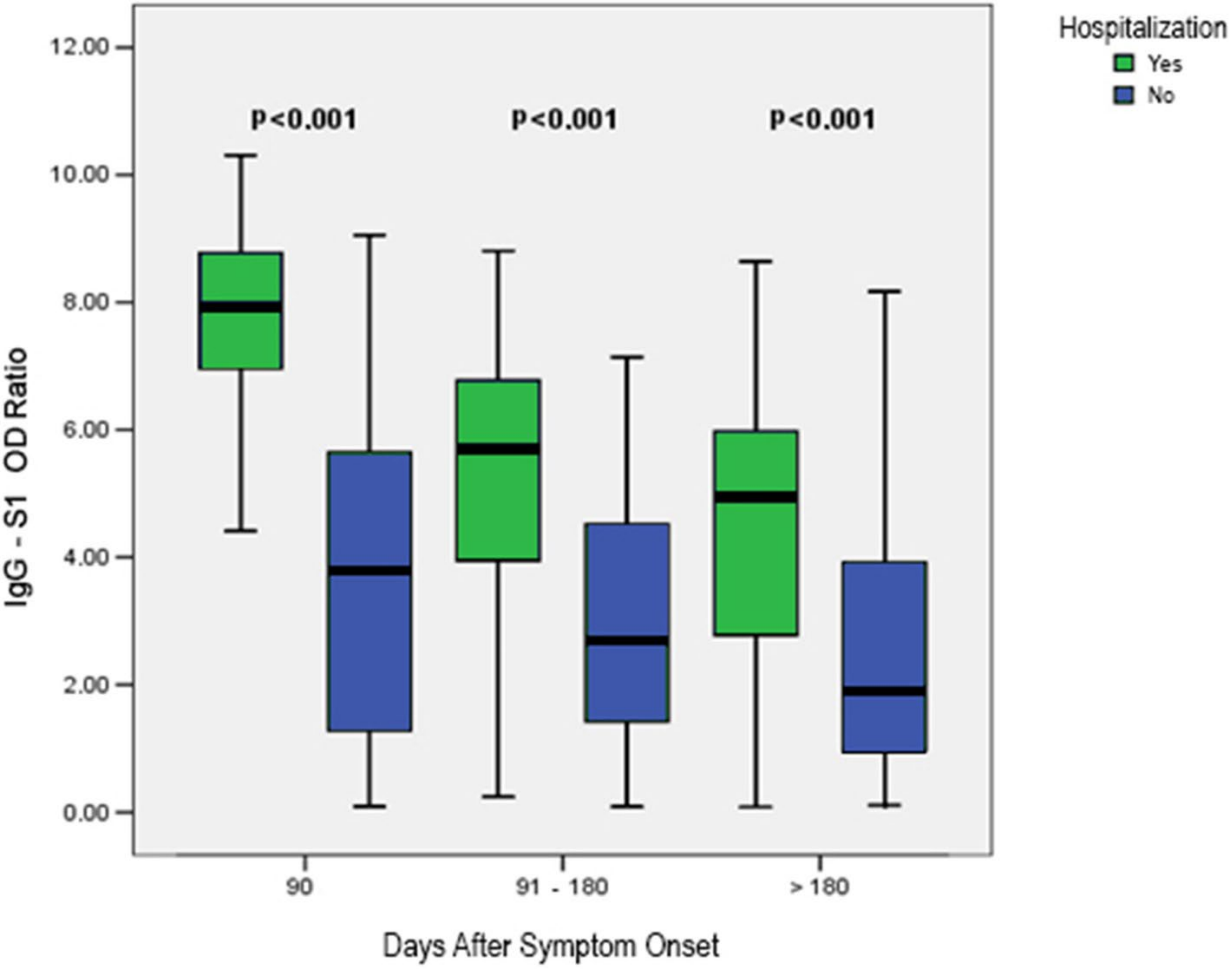
Antibody IgG-S1 serum titers in 238 individuals who recovered from COVID-19 according to hospitalization at 386 days after primary infection. A comparison of the median level of serum IgG-S1 antibodies between samples from the Mild Group (blue box) and the Severe Group (green box) demonstrated higher serum IgG-S1 titers among patients from the Severe Group at all collection times analyzed over the 386 days A drop in serum IgG-S1 titers was observed in both groups during the follow-up period of the study. OD ratio: denotes ratio between the absorbance level of the patient’s sample by the absorbance level of the calibrator

A comparison of the median level of serum IgG-S1 antibodies between samples from the Mild Group and the Severe Group demonstrated higher serum IgG-S1 titers among patients from the Severe Group at all collection times analyzed over the 386 days (p < 0.001). On the other hand, a drop in serum IgG-S1 titers was observed in both groups, despite the persistence of IgG-S1 during the follow-up period of the study (Fig. 2). Significantly, higher levels of IgG-S1 were observed in samples collected up to 90 days after symptom onset compared to samples collected 90 days and 180 days after symptom onset (Table 2).

**Table 2:** Factors that impact the serum level of IgG-S1 antibodies against SARs-CoV-2 in samples of individuals after recovering from the first infection of COVID-19

Variables	IgG Antibody	*p*-value
	**Median (Q1; Q3)**	
**Hospitalization**		
Yes	6.76 (4.98; 8.14)	< 0.001*
No	2.67 (1.18; 4.74)	
**Sex**		
Male	4.95 (1.73; 7.33)	0.033*
Female	3.70 (1.90; 6.07)	
**Obesity**		
Yes	4.96 (2.49; 6.81)	0.014*
No	3.62 (1.47; 6.78)	
**Symptoms at diagnosis**		
1–3	3.92 (1.32; 6.70)	0.026**
4–5	5.02 (2.67; 7.38)	
≥ 6	4.16 (1.82; 6.31)	
**Age (in years)**		
18 to 45	2.20 (1.01; 4.08)	< 0.001**
46 to 59	5.33 (2.60; 6.98)	
60 or over	5.65 (3.19; 7.45)	
**SAH**		
Yes	5.85 (3.26; 7.42)	< 0.001*
No	3.27 (1.42; 5.83)	
**T2DM**		
Yes	5.97 (3.94; 7.64)	< 0.001*
No	3.81 (1.58; 6.29)	
**IgG antibody titers in DASO**		
Up to 90 DASO	6.20 (3.43; 8.08)	< 0.001**
91 to 180 DASO	3.82 (2.05; 6.10)	
> 180 DASO	2.63 (1.25; 5.01)	

(*) Mann–Whitney (**) Kruskal–Wallis

SAH denotes Systemic arterial hypertension, T2DM Type 2 diabetes mellitus, DASO Days after symptom onset, IgG-S Anti-spike Immunoglulin G

This tables show the results of Mann-Withney and Kruskal–Wallis to indicate the impact of each variable separately on the serum level IgG-S1 antibodies against SARs-CoV-2

**Fig. 3:**
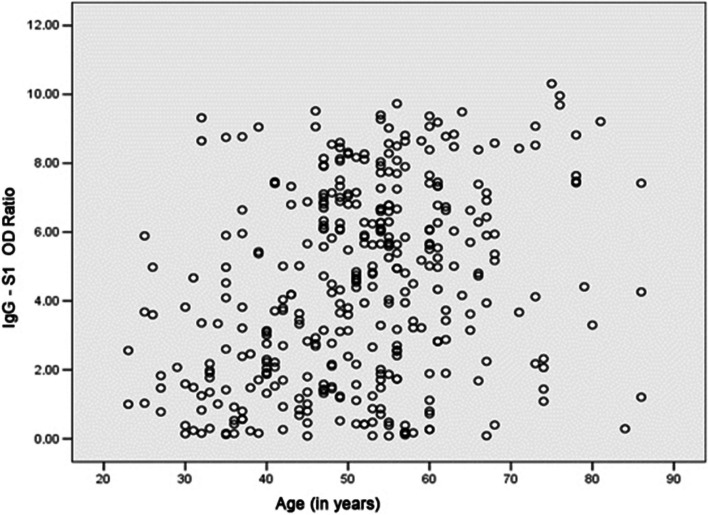
Spearman correlation coefficient between serum titers according to age = 0.298. Although there was a weak correlation between increasing age and humoral IgG-S1 response (Spearman correlation = 0.298), we noted that individuals aged over 45 years presented significantly higher median levels of IgG-S1 when compared to younger individuals (Table 2). OD Ratio: denotes ratio between the absorbance level of the patient's sample by the absorbance level of the calibrator

**Fig. 4:**
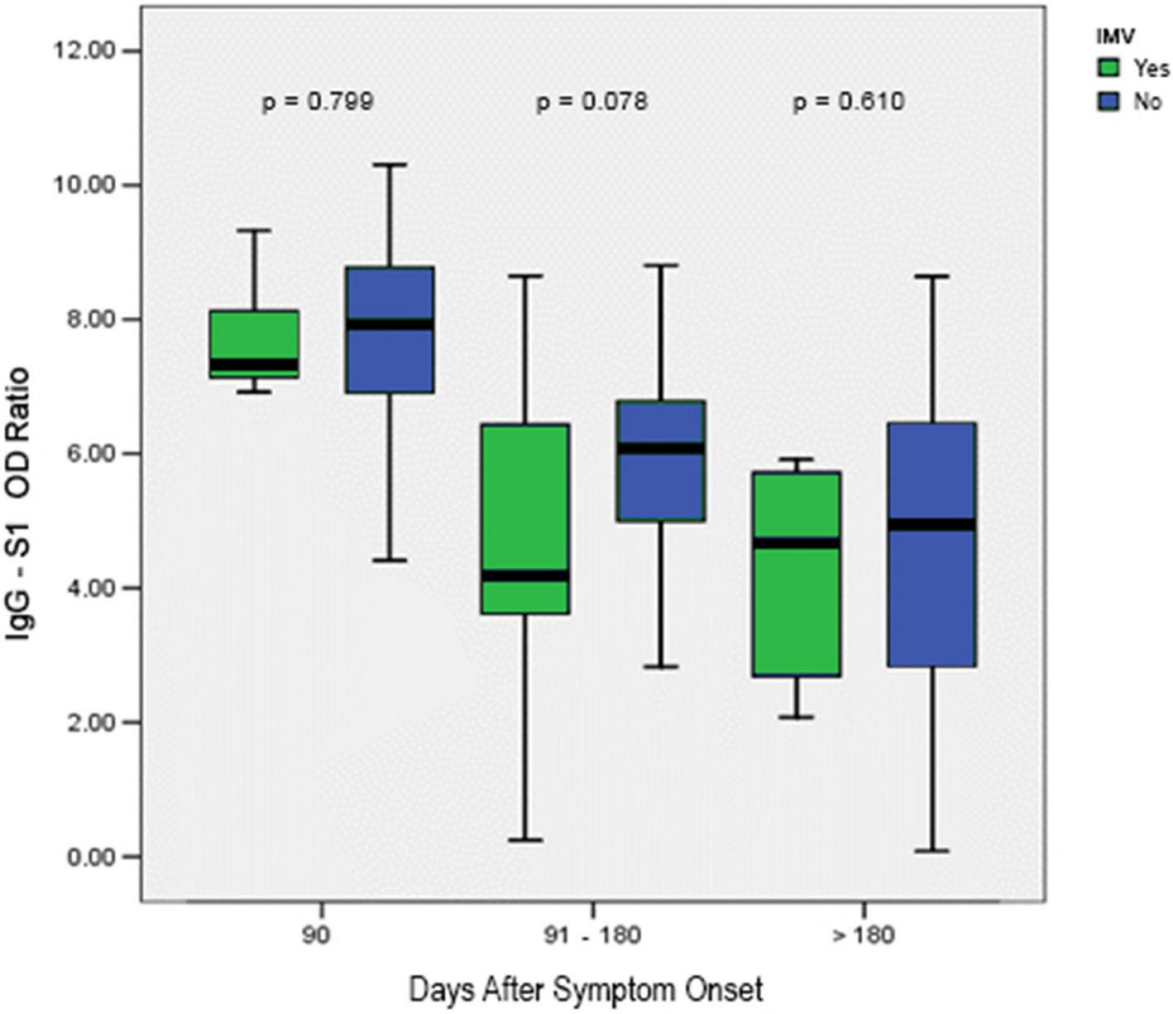
Antibody IgG-S1 serum titers in 87 hospitalized individuals who recovered from COVID-19 according to oxygen demand (invasive and non-invasive oxygen therapy). There was no statistically significant difference between the samples of participants who received invasive and non-invasive oxygen therapy support at all intervals of days after the onset of the analyzed symptoms

Higher levels of IgG-S1 were observed in male, obese, hypertensive individuals with T2DM and individuals with four or more symptoms at the time of diagnosing COVID-19 (Table 2). Although there was a weak correlation between increasing age and humoral IgG-S1 response (Spearman correlation = 0.298; Fig. 3), we noted that individuals aged over 45 years presented significantly higher median levels of IgG-S1 when compared to younger individuals (Table 2). On the other hand, there was no statistically significant difference between the samples of participants who received invasive and non-invasive oxygen therapy support at all intervals of days after the onset of the analyzed symptoms (Fig. 4).

**Fig. 5:**
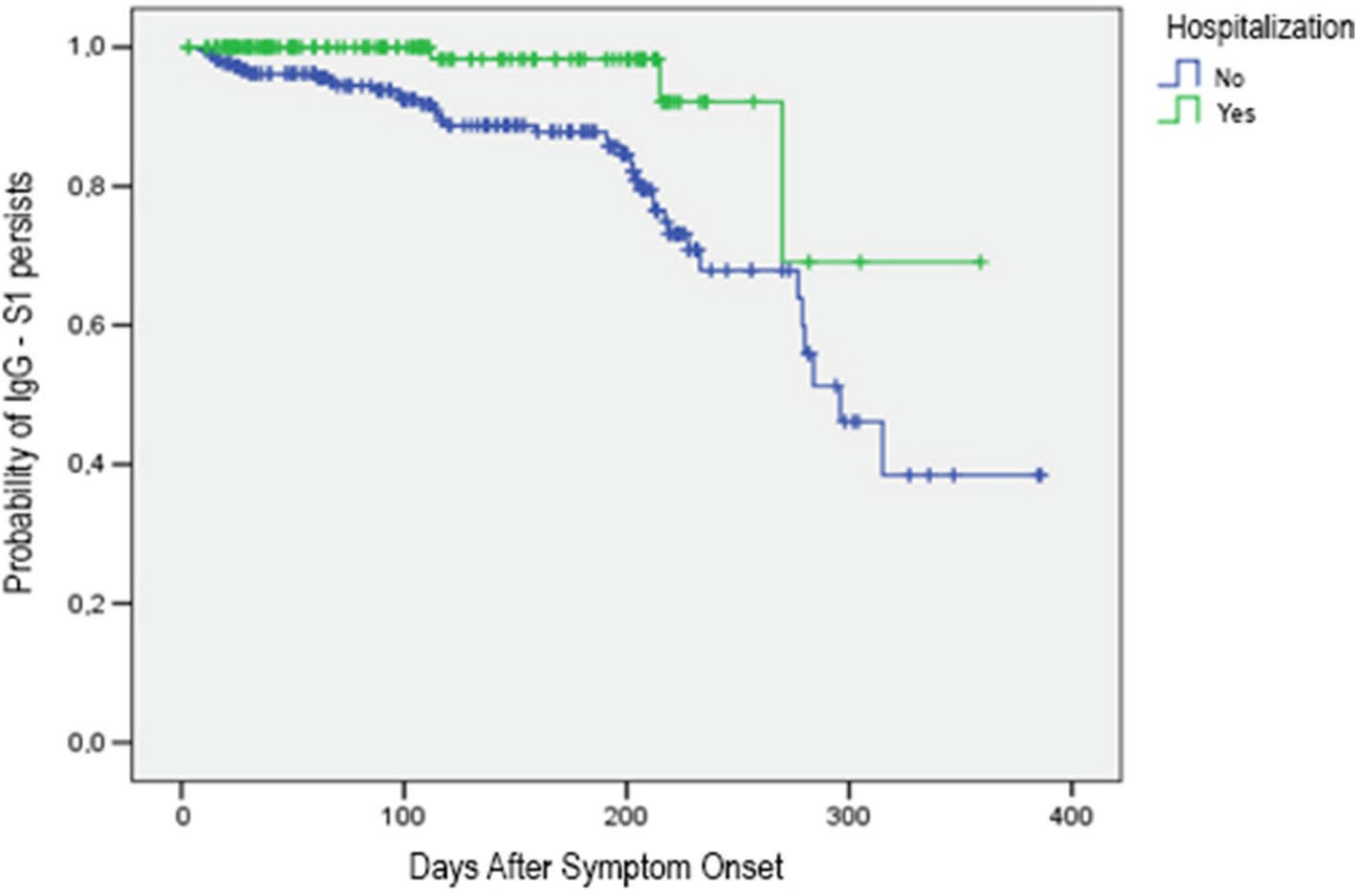
Probability of IgG-S1 antibodies persisting according to severity in 238 individuals who recovered from COVID-19 over 386 days. (Kaplan Meier Curve: Log Rank (Mantel-Cox): Chi-Square 10.1575 df 1 *p* = 0.001 Testing equality of survival distributions for the different levels of Hospitalization.). There was a greater probability of IgG-S1 antibody persisting over time among samples from hospitalized individuals when compared to samples from non-hospitalized participants (*p* = 0.001)

There was a greater probability of IgG-S1 antibody persisting over time among samples from hospitalized individuals when compared to samples from non-hospitalized participants (*p* = 0.001) (Fig. 5). Samples with IgG-S1 antibody titers below the cut-off point (negative) were more frequent in the non-hospitalized group, in those younger than 60 years and in those with no comorbidities.

In the multivariate analysis (Table 3), only hospitalization, age, number of symptoms at the time of diagnosis and the time of sample collection after the onset of symptoms continued to impact the humoral response. The severity of COVID-19 manifested the highest regression coefficient. It is estimated that, on average, the need for hospitalization increases the serum IgG-S1 titers by 2.91 units (coefficient 2.91, *p* = 0.001).

**Table 3:** IgG-S1 antibody model

Variables	Coefficients		*p*-value
	**Unstandardized**	**Standardized**	
Constant	2,41	–-	< 0,001
Days after symptom onset	-0,01	-0,24	< 0,001
Age (in years)	0,02	0,09	0,044
Symptoms at diagnosis	0,14	0,10	0,014
Hospitalization	2,91	0,51	< 0,001

Adjusted R Squared: 0.417

This table shows the multivariate analysis that included variables that attained a level of p < 0.2 in the univariate analysis. They were entered in a linear regression model using the Enter method

Variables that began in the model: Sex, Obesity, SAH, T2DM, Hospitalization, Days after symptom onset, Age (in years) and number of symptoms. The severity of COVID-19 manifested the highest regression coefficient. SAH denotes Systemic arterial hypertension, T2DM Type 2 diabetes mellitus, DASO Days after symptom onset

Our results presented higher median IgG-S1 antibody titers in the Severe Group samples in all the studied time intervals. Several studies have previously established the association between the severity of COVID-19 and the magnitude of the humoral response in the first four months after the onset of symptoms [14–16]. Our results also demonstrate that this association remains over twelve months. We believe that a higher viral and antigenic load in severe individuals [18–20] elicit a greater IgG-S1 antibody response and thereby justify the findings of our study. However, it is still controversial as to whether higher viral loads are associated with poorer outcomes in COVID-19 [21–24].

In the univariate analysis, hospitalization seemed to have an equal impact as the other variables on serum IgG-S titers. After multivariate analysis, it was observed in the final model that the variable with the greatest influence on the serum level of IgG-S1 antibodies was hospitalization, i.e., the severe presentation of COVID-19.

Despite the persistence of IgG-S1, there was a decrease in the antibody titers in both groups over the follow-up period. A negative association was observed between the variable days after symptom onset and the IgG-S1 antibody levels, suggesting that as time passes, the IgG-S1 antibody titer decreases in peripheral blood. A similar outcome to this has been reported in other studies [13, 25]. This dynamic of the humoral response in COVID-19 is analogous to many other viruses, including those that induce lifelong immunity, such as measles, experiencing a contraction phase [26, 27].

We observed a higher frequency of seronegativity and a greater probability of seronegativity for IgG-S1 during follow-up in the Mild Group. This finding was also reported by a study, which observed that 40% of asymptomatic individuals and 12% of symptomatic individuals presented with negative serology after a 90-day period [6]. While it is assumed that the risk of reinfection in this population is higher, it is not possible, however, to draw this conclusion, and more studies are required. A retrospective study reported no difference in the severity of COVID-19 when comparing individuals with the presence of IgG antibodies and individuals with no IgG antibodies [28].

In our sample, no association was observed between age and serum IgG-S1 titers in the final model. The link between age and antibodies in COVID-19 is controversial. However, advanced age was a predictive factor for hospitalization, in line with a number of other studies [29, 30]. It is known that immunosenescence promotes quantitative changes in cells and components of the immune system, together with more complex changes in the action of several immune responses. Older people are less able to respond effectively to new antigens, in addition to chronic low-grade inflammation brought about by an increase in the production of pro-inflammatory cytokines, acute-phase proteins and oxidative stressors [31].

Corroborating our findings, being male has been described as a risk factor for hospitalization [32, 33], but without influencing serum antibody titers. Gender differences in the immune response to COVID-19 have been described. Males present a pro-inflammatory response at the expense of IL-6, IL-8, GROα, sCD4L, MIP-1β, MCP-1. Some studies have demonstrated higher levels of the ACE2 receptor, used for viral entry into target cells, in males. The cellular serine protease TMPRSS2, responsible for activating the spike (S) protein of the coronavirus, is also highly expressed in the prostate epithelium and is sensitive to androgens [34]. However, in the present study, no difference was observed in serum IgG-S antibody titers between genders.

As with the variable sex, comorbidities such as SAH and T2DM demonstrated no association with the humoral response after adjusting for the other variables. They were more frequent in the Severe Group, thereby demonstrating a connection with the criticality of COVID-19. The link between SAH and the IgG-S antibody response has not been studied. There is controversy surrounding the hypothesis that hypertension is an independent predictor of severity [35].

The combination of SAH with T2DM or another comorbidity may be more relevant as a predictor of severe COVID-19 [36]. On the other hand, it is well established that T2DM and hyperglycemia increase the chance of hospitalization, but do not represent a decrease in serum levels of IgG-S antibodies after primary infection with SARS-CoV-2 [37], which is in agreement with our findings. Individuals with T2DM have an impaired proliferative response of lymphocytes, as well as disorders of the monocytes, macrophages, and neutrophils and in the complement activation [38].

In the present study, obesity, after the multivariate analysis, demonstrated no impact on the serum levels of the IgG-S antibody. However, Frasca et al. reported lower anti-IgG-S antibody titers in individuals with obesity, and a body mass index negatively associated with serum levels of IgG-S1 antibodies in COVID-19 [39]. These findings are consistent with the knowledge that obesity is associated with chronic low-grade inflammation and, in turn, a dysfunctional immune system [40, 41].

Although it is not a viral neutralization test, the semiquantitative EUROIMMUN IgG-S1 Kit was described as having a strong correlation with the neutralization test [42]. It is important to note that the small number of asymptomatic patients was due to the difficulty in finding these patients in the first wave of the pandemic, when tests for COVID-19 were scarce in Brazil and were reserved only for those with symptoms.

Other limitations included the fact that most study subjects only provided a single blood sample, and there was no continuous follow-up. Additionally, the study solely analyzed IgG-S1 antibodies. Due to limited resources, it was not possible to include tests for IgA and IgM antibodies, as well as against targets other than the spike protein.

## Conclusions

In sum, this study has revealed higher antibody titers and a higher likelihood of IgG-S persisting in severe cases after SARS-CoV-2 primary infection in unvaccinated patients who have recovered. Thus, in this study, the severe clinical presentation of COVID-19 was the main factor influencing serum levels of IgG-S1 antibodies in COVID-19.

## Data Availability

Data are available upon request from the corresponding author.

## Abbreviations

*SARS-CoV-2*: Severe acute respiratory syndrome-coronavirus
*COVID-19*: Coronavirus disease 2019
*SARS*: Severe acute respiratory syndrome
*ARDS*: Acute respiratory distress syndrome
*IgG*: Immunoglobulin G
*IgG-S*: Anti-spike Immunoglobulin G
*RT-PCR*: Real-time polymerase chain reaction
*LACEN-PE*: Central Laboratory of Pernambuco
SAH: Systemic arterial hypertension
*T2DM*: Type 2 diabetes mellitus
